# Prevalence of alpha and beta haemolysin among blood group O donors in Bamenda, Cameroon

**DOI:** 10.4102/ajlm.v11i1.1432

**Published:** 2022-04-19

**Authors:** Victor N. Fondoh, Nobert Ndzenjempuh, Tamunjoh Stella, Richard M. Fondoh, Charles N. Awasom, Rebecca Enow-Tanjong, Egbe P. Egbengu, Robert Leke, Njini F.N. Rose, Denis Nsame

**Affiliations:** 1 Bamenda Regional Hospital Laboratory, Regional Hospital Bamenda, Cameroon; 2 Department of Medical Laboratory Sciences, School of Health and Medical Sciences, Catholic University of Cameroon, Bamenda, Cameroon; 3 Department of Health Economics Policy and Management, Faculty of Business Management, Catholic University of Cameroon, Bamenda, Cameroon; 4 North-West Regional Fund for Health Promotion, Bamenda, Cameroon; 5 Department of Anatomy, School of Health and Medical Science, Catholic University of Cameroon, Bamenda, Cameroon; 6 Department of Medicine and Surgery, School of Health and Medical Science, Catholic University of Cameroon, Bamenda, Cameroon; 7 Regional Hospital Bamenda, Bamenda, Cameroon

**Keywords:** prevalence, haemolysin, immunoglobulin, blood group O, donors, Bamenda, Cameroon

## Abstract

**Background:**

The occurrence of high titres of alpha (anti-A) and beta (anti-B) haemolysin immunoglobulin G antibodies in blood causes haemolysis during blood transfusion from a group O donor, commonly and inappropriately known as the ‘universal blood donor’, to a group A, B or AB recipient. Surprisingly, haemolysin testing is not routinely done during blood transfusion services in Bamenda, Cameroon.

**Objective:**

This study aimed to determine the prevalence of haemolysin among blood group ‘O’ donors at the Regional Hospital Bamenda Blood Bank, Bamenda, Cameroon.

**Methods:**

This was a cross-sectional descriptive study carried out between June and September 2020 at the Regional Hospital Bamenda Blood Bank, Bamenda, Cameroon. Blood group O donors who were free from transfusion-transmissible infections were selected systematically and serially and their serum tested for the presence of haemolysin. Haemolysin titres were determined, and titres ≥ 8 were considered significant. The associations between haemolysin prevalence and age group, gender and Rhesus D blood group were determined using the chi-square test.

**Results:**

The prevalence of haemolysin among the 480 study participants was 52.1% and significant haemolysin titres were detected in 18.5%. There was no association between haemolysin and gender, age group or the Rhesus D blood group.

**Conclusion:**

The prevalence of significant titres of haemolysin among participants in this study was high. There is the need to test for haemolysin in blood group O donors to prevent the potential risk to blood group A, B, and AB recipients and to provide safer blood for transfusion.

## Introduction

The occurrence of alpha (anti-A) and beta (anti-B) immunoglobulin M antibodies in the absence of corresponding red blood cell antigens is a significant feature of the ABO blood group system in individuals.^1^ When blood transfusion is done without consideration for ABO compatibility, these naturally occurring antibodies are a potential cause of dangerous haemolysis in recipients.^2^ Although these antibodies react optimally at 4 °C, they may also cause haemolysis at 37 °C. Also, some blood group O, and sometimes blood group A_2_, individuals may develop alpha and beta antibodies of the immunoglobulin G class that react optimally at 37 °C. These antibodies are commonly referred to as haemolysins and are more dangerous compared to naturally occurring haemolysins.^2^ In cases of non-iso group-compatible ABO transfusion, these antibodies can trigger the complete complement cascade, leading to haemolytic reactions.^2^ Several studies have reported complications in patients who were transfused with non-identical blood groups, including disseminated intravascular coagulation,^3,4^ hepatic and renal failure leading to death,^3,5^ paleness, jaundice, fever, ecchymosis and generalised exfoliation of the skin,^6^ significant reduction in packed cell volume,^7^ hyperbilirubinaemia,^8^ varying degrees of haemoglobinaemia, and intravascular agglutination.^9^ Several studies have advocated that the transfusion of group O blood to A, B, or AB recipients be discontinued due to the high prevalence of alpha and beta haemolysins among blood group O donors.^10,11,12^ Despite this concern, the practice is yet to be discontinued.

Several studies have postulated that these alpha and beta antibodies originated as products of immunisation from allogenic stimulation (due to transfer of antigen by red cells from pregnancy with an ABO-incompatible foetus, incompatible blood transfusion, tissue transplant, etc.) and heterogeneous stimulation (due to vaccination, serotherapy, and inoculation from vectors such as blood-sucking insects and certain pharmaceutical preparations contaminated with alpha- and beta-like antigens, etc.).^13,14,15^ Due to the high demand for and shortage of allogenic blood in most developing countries, including Cameroon, as well as the relative difficulty in getting ABO group-compatible blood during emergencies, blood group O, which is often inappropriately referred to as the ‘universal blood donor’, has been increasingly transfused to non-group O recipients.^16^ Besides, there is evidence that blood group O is the most abundant group in the ABO blood system, with a prevalence of 51.1% reported in a study in Cameroon.^17^

Blood transfusion is an essential medical practice that replenishes lost blood or blood products in the recipient. The transfused blood should be as safe as possible to ensure maximum benefit to the recipients. Thus, there is the need to strictly adhere to standard screening protocols to achieve safe blood transfusion. Unfortunately, this practice is limited in the developing world due to inefficient blood banking systems and scarcity of screening facilities. Besides, the standard protocol for compatibility testing to ensure safe blood has been omitted or abbreviated by many screening services. This standard compatibility testing protocol^18^ requires that all group O blood intended for transfusion to group A, B, or AB recipients should be screened for the presence of high titres of alpha and beta haemolysins and that only haemolysin-free blood should be reserved for blood group A, B, or AB recipients, while haemolysin-positive blood should be reserved for group O recipients only.^13^ Several countries, including Côte d’Ivoire, have included a haemolysin test as part of the standard protocol in their national blood transfusion programmes.^18^ Surprisingly, this practice is yet to be implemented in blood bank facilities in Bamenda or Cameroon in general. Hence, this study aimed to determine and demonstrate the presence of significant titres of alpha and beta haemolysins among blood group O donors at the Regional Hospital Bamenda Blood Bank (RHBBB), Bamenda, Cameroon, to guide the implementation of a policy to include haemolysin testing in the protocol for compatibility testing in Cameroon.

## Methods

### Ethical considerations

Administrative authorisation to carry out this work was provided by the Catholic University of Cameroon, Bamenda, North-West Regional Delegation of Public Health, Bamenda, and Regional Hospital Bamenda. Ethical clearance was provided by the Institutional Review Board of the Regional Hospital Bamenda (IRB number: 211/APP/RDPH/RHB/IRB). Participants provided written informed consent and were free to withdraw from the study at any time. The participants’ data were coded by assigning numbers to identify the participants instead of names. The anonymity of participants and their data were ensured by storing the data on password-protected computers and in locked file cabinets accessible only to the study staff and researchers.

### Study area

This research was carried out in Bamenda at the RHBBB, Bamenda, a unit at the Regional Hospital Bamenda. The RHBBB receives approximately 5400 blood donors and issues about 4200 safe pints of blood yearly. It has a standard blood transfusion service and is enrolled in a certification programme with the Safe Blood for Africa Foundation. It is also the largest blood transfusion centre in the North-West region and provides transfusion services to the region and beyond.

### Research design

This was a descriptive, cross-sectional study conducted between June 2020 and September 2020 at the RHBBB. The sample size was calculated based on a previous study conducted in 2011 in Eastern Nigeria^16^ that reported an overall haemolysin prevalence of 55.4%. A minimum of 385 participants was required for this study.

Blood donors arriving at the reception area of the RHBBB undergo routine screening to determine physical fitness to donate blood using a standard questionnaire validated and provided by the RHBBB quality team. This routine screening selects individuals who had not donated blood and had no history of sexually transmitted diseases in the three months preceding the blood donation, were free from non-communicable diseases such as diabetes and hypertension, had not been vaccinated in the last four months, had not taken medication for at least one week, and had not smoked on the day of the donation or taken alcohol in the last 24 h. Women who were pregnant, breastfeeding, or menstruating or expecting their menses within one week were excluded. In addition, only donors who weighed greater than 50 kg, were between the ages of 18 and 60 years (women) or 18 and 65 years (men) and had blood pressures between 100 mmHg and 140 mmHg over 60 mmHg – 100 mmHg and temperatures between 36 °C and 37.5 °C were endorsed as fit for blood donation.

As part of the routine protocol for screening donors to obtain safe blood in the blood bank, ABO and Rhesus D blood group tests and transfusion transmittable infection (TTI) tests were done on samples from all donors. ABO and Rhesus blood groups were determined using the procedure described by Dacie and Lewis^19^ using the blood in the ethylenediaminetetraacetic acid tube. The TTI test was done using the blood in the plain tube. TTI testing included the following: HIV test using the HIV-1/2 Ag/Ab Combo Determine (Alere Medical Co., Ltd, Matsuhidai, Matsudo-Shi, Chiba-ken, Japan) as the first-line test and OraQuick (OraSure Technologies, Inc., Bethlehem, Pennsylvania, United States) as the second-line test; hepatitis B and hepatitis C virus tests using the DIASpot diagnostic kit (DIASpot Diagnostics, Jawa Barat, Indonesia); syphilis test using the rapid plasma reagin (RPR)-carbon slide agglutination assay (Cypress Diagnostics, Langdorp, Belgium) and *Treponema pallidum* haemagglutination assay (Omega Diagnostic, Alva, Scotland, United Kingdom); and malaria test using the CareStart^TH^ Malaria pf/PAN (HRP2/pLDH)Ag Combo RDT (AccessBio, Somerset, New Jersey, United States).

As part of the donation process, blood samples were collected into two tubes – one in a plain tube and the other in an ethylenediaminetetraacetic acid tube from donors already screened using the questionnaire as fit for blood donation.

The screened donors were systematically and serially contacted to participate in the study. Only donors who were blood group O, free from all the TTIs, and who consented to be part of the study were included. A standard data collection format was used to collect information on the age, blood groups and gender of the study participants.

### Haemolysin test and titration

Blood specimens collected in the plain tubes (used for TTI screening) were used to determine haemolysin titres within 24 h of specimen collection. Briefly, the sample was allowed to clot for about 45 min and then centrifuged to separate the serum. The serum was then tested for the presence of haemolysins.^11,16^

Zero point 5 mililetres of the serum was placed in three test tubes labelled ‘A’, ‘B’ and ‘O’, and 0.5 mL of 5% blood group A, B, or O washed red cells suspended in physiological saline was added to each tube. The blood group O red cells were used as a negative control. The setup was incubated at 37 °C for 2 h and centrifuged afterwards. The supernatant was then examined macroscopically (in bright light) and microscopically for the presence of haemolysins. The degree of haemolysis was graded as 1+ for traces of haemolysis, 2+ for partial (greater than 50% but not complete) haemolysis, 3+ for complete haemolysis, and negative when no haemolysis was observed.^11,20,21,22^

All sera positive for haemolysis were titrated to quantify the degree of haemolysis.^11,20,21^ 0.5 mL of the sera was diluted twofold with physiological saline to a titre of 526, and 0.5 mL of 5% washed red cells of the respective positive sera was added. The setup was incubated for 2 h and observed for haemolysis macroscopically and microscopically. The reciprocal of the serum dilution in the last tube with haemolysis was considered as the titre.^11,20,21^

### Statistical analysis

Collected data were entered into Microsoft Excel 2010 (Microsoft Corporation, Redmond, Washington, United States) and double-checked for errors by a second person. All analyses were done using Statistical Package for Social Sciences version 20 (IBM Corp., Chicago, Illinois, United States). Haemolysin prevalence was determined as the proportion of participants whose blood samples were positive for haemolysin (alpha, beta or both alpha and beta haemolysin). Haemolysin titres equal to or greater than 8 were considered significant. The prevalence of significant titres was also determined as the proportion of participants with significant haemolysin titres. Associations between haemolysin prevalence and age group, gender and Rhesus D blood group were determined using chi-square test, and *p*-values < 0.05 were considered as statistically significant.

## Results

**FIGURE 1:**
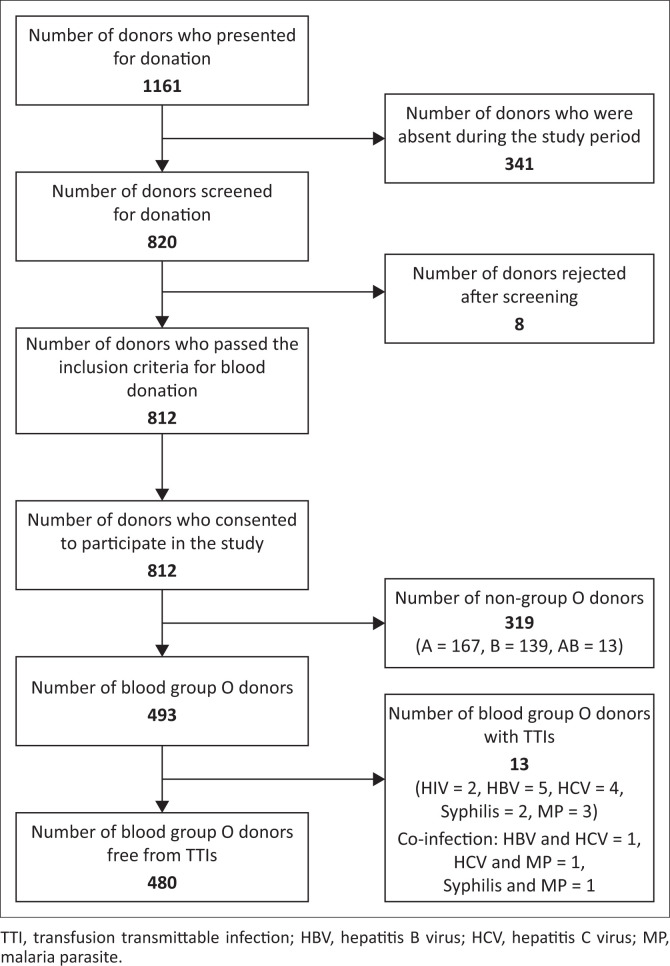
Selection of study participants among blood group O donors at the RHBBB, Cameroon, June 2020 – September 2020.

In total, 1161 blood donors presented to the RHBBB for blood donation between June 2020 and September 2020, 820 of whom were screened for physical fitness. Of the 820 donors, 812 were classified as physically fit based on the applied standard questionnaire prepared by the RHBBB, and all the 812 donors consented to participate in the study. Out of the screened 812 donors, 493 were blood group O, 480 of whom were free from TTIs and were included to participate in the study (Figure 1).

Participants were aged between 18 years and 55 years and comprised 383 (79.8%) men and 97 (20.2%) women (Table 1). Four hundred and sixty-three (96.5%) of the participants were Rhesus D positive and 17 (3.5%) were Rhesus D negative. Of the 480 blood group O donors tested for haemolysins, 230 were negative while 250 were positive, giving a haemolysin prevalence of 52.1%. Haemolysins were detected in 204 (53.3%) men and 46 (47.4%) women. The single participant aged ≥ 55 years was positive for haemolysin. In the other age groups, haemolysin prevalence was highest among participants aged between 45 and 54 years (28/48; 58.3%), followed by participants aged 18–24 years (86/156; 55.1%), 35–44 years (44/87; 50.6%) and 25–34 years (91/188; 48.4%). There was no association between haemolysin production and gender (*p* = 0.304), age group (*p* = 0.501) or Rhesus D positivity (*p* = 0.628). Two hundred and forty (240) of the 463 Rhesus D-positive participants (51.8%) were positive for haemolysin while 10 (58.8%) of the 17 Rhesus D-negative participants were positive for haemolysin.

**TABLE 1:** Association between haemolysin production and gender, age group or Rhesus D positivity of blood group O donors at the RHBBB, Cameroon, June 2020 – September 2020.

Haemolysin production	Total (*N* = 480)		Negative (*n* = 230)		Positive (*n* = 250)		*p*
	*N*	%	*n*	%	*n*	%	
**Gender**							
Male	383	79.8	179	46.7	204	53.3	0.304
Female	97	20.2	51	52.6	46	47.4	
**Age group**							
18–24	156	32.5	70	44.9	86	55.1	0.501
25–34	188	39.2	97	51.6	91	48.4	
35–44	87	18.1	43	49.4	44	50.6	
45–54	48	10.0	20	41.7	28	58.3	
> 55	1	0.2	0	0.0	1	100.0	
**Rhesus D**							
Positive	463	96.5	223	48.2	240	51.8	0.628
Negative	17	3.5	7	41.2	10	58.8	

**FIGURE 2:**
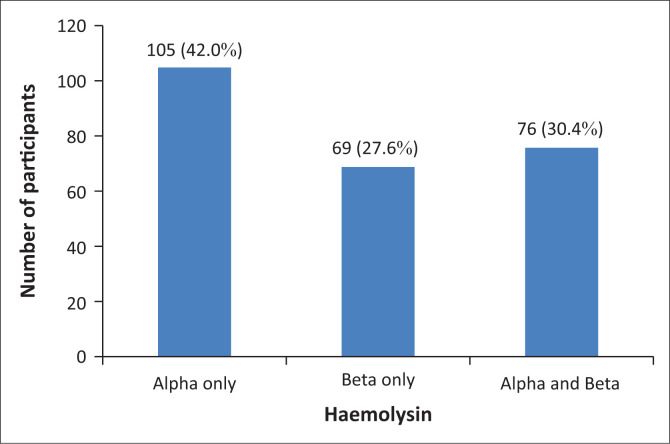
The prevalence of alpha and beta haemolysins among haemolysin-positive blood group O donors at the RHBBB, Cameroon, June 2020 – September 2020.

**FIGURE 3:**
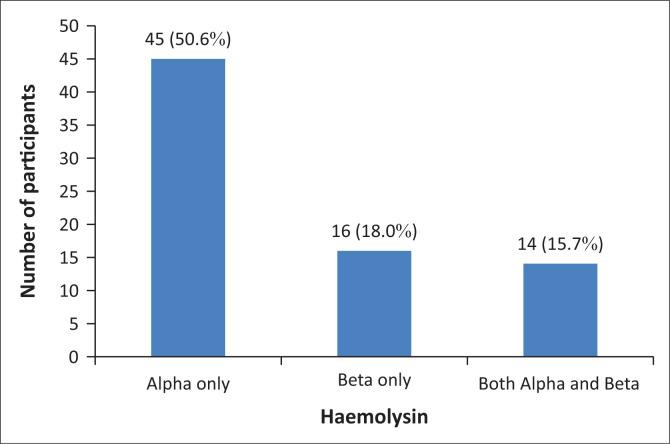
Prevalence of significant titres of alpha and beta haemolysins among blood group O donors at the RHBBB, Cameroon, June 2020 – September 2020.

Of the 250 participants positive for haemolysin (alpha, beta or both), 105 (42.0%) were positive for only alpha haemolysin, 69 (27.6%) were positive for only beta haemolysin, and 76 (30.4%) were positive for both alpha and beta haemolysins (Figure 2). Haemolysins from the 181 participants positive for alpha haemolysin showed trace haemolysis (47; 26.0%), partial haemolysis (95; 52.5%) and complete haemolysis (39; 21.5%) (Table 2). Of the haemolysins from the 145 participants positive for beta haemolysin, 58 (40.0%) showed trace haemolysis, 58 (40.0%) showed partial haemolysis, and 29 (20.0%) showed complete haemolysis. The highest observed haemolysin titre was 32 and was detected in five (2.8%) alpha haemolysin-positive participants and three (2.1%) beta haemolysin-positive participants. Eighty-nine (89; 18.5%) participants presented with significant haemolysin titres (Table 3), of which 45 (50.6%) were alpha haemolysin-positive only, 16 (18.0%) were beta haemolysin-positive only, and 14 (15.7%) were positive for both alpha and beta haemolysin (Figure 3).

**TABLE 2:** Degree of haemolysis of alpha and beta haemolysins from blood group O donors at the RHBBB, Cameroon, June 2020–September 2020.

Degree of haemolysis	Total (*N* = 326†)		Alpha (*n* = 181)		Beta (*n* = 145)	
	*N*	%	*n*	%	*n*	%
Trace	105	32.2	47	26.0	58	40.0
Partial	153	46.9	95	52.5	58	40.0
Complete	68	20.9	39	21.5	29	20.0

† , This number is greater than the total number of participants because some of the participants were positive for both alpha and beta haemolysin and were counted twice.

**TABLE 3:** Titres of alpha and beta haemolysins among blood group O donors at the RHBBB, Cameroon, June 2020–September 2020.

Titres	Total (*N* = 326†)		Alpha (*n* = 181)		Beta (*n* = 145)	
	*N*	%	*n*	%	*n*	%
1	68	20.9	27	14.9	41	28.3
2	73	22.4	46	25.4	27	18.6
4	96	29.4	49	27.1	47	32.4
8	51	15.6	33	18.2	18	12.4
16	30	9.2	21	11.6	9	6.2
32	8	2.5	5	2.8	3	2.1

† , This number is greater than the total number of participants because some of the participants were positive for both alpha and beta haemolysin.

## Discussion

This study was carried out to determine the prevalence and titres of haemolysin among blood group O donors at the RHBBB. Our study found a high haemolysin prevalence of 52.1% among the study population. Significant haemolysin titres (defined as titres ≥ 8) were also detected in a high proportion (18.5%) of participants. This high prevalence may be attributed to immunisation arising from exposure to mosquito bites and parasitic infections of the gastrointestinal system.^22^ High prevalence rates of malaria^23^ and gastrointestinal parasites^24^ have been reported in Bamenda, North-West Region, Cameroon.^25^ The high prevalence of haemolysin in this study is comparable to that (52.8%) reported by a study in Abakaliki, Nigeria, in 2014.^25^ Lower haemolysin prevalence rates have been reported by studies among healthy blood donors in South India (10.8% in 2019),^26^ Abidjan, Côte d’lvoire^18^ (35.1% in 2016), Lagos, Nigeria^10^ (30.3% in 2015), Bauchi, Nigeria^27^ (22.2% in 2015), Anambra, Nigeria^28^ (16.06% in 2015), Tunisia^14^ (6.6% in 2008), Ilorin, Nigeria^11^ (23.2% in 2001), and Nigeria^12^ (30.6% in 1990). A higher prevalence of 69.0% was reported in a 2012 study on healthy blood donors in Bangkok, Thailand.^29^ These differences in prevalence rates may be due to the admixture of blood of immigrants as a result of intermarriages,^20^ variations in serum-cell ratios,^30^ or differences in geographical location,^26^ particularly due to the differences in the degree of exposure to gastrointestinal parasites^22^ and mosquitoes.^13,14,15^ It has been reported that higher serum-cell ratios increase the tendency for red cell lysis.^30^

The alpha haemolysin prevalence in this study (53.6%) was higher than that reported in a study conducted in 2010 in Southeast Nigeria that reported a prevalence of 10.3% for alpha haemolysin.^20^ In contrast, the observed prevalence rates of beta haemolysin, and both alpha and beta haemolysins in this study were lower than that reported in the same study (8.3% vs 12.6% for beta haemolysin, and 15.8% vs 32.5% for both alpha and beta haemolysins).^20^ Alpha haemolysins were more prevalent in our study compared to beta haemolysin, which is consistent with the findings of a study conducted in 2015^11^ in Lagos, Nigeria, but different from the findings of another study conducted in 2001 in Ilorin, Nigeria, that observed a higher prevalence of beta haemolysin compared to alpha haemolysin.^11^ The reasons for these variations may either be genetic or environment-induced.^31^

The absence of associations between haemolysin production and gender, age group or Rhesus D blood group of blood group O donors in our study is consistent with the findings from previous studies.^4,10,11,14,18,31,32^

The prevalence of significant haemolysin titres (titres ≥ 8) in our study is noteworthy, considering the evidence that titres above this threshold can significantly cause haemolysis in vivo.^33^ This may be because parasitic infections such as malaria are endemic in the study area. There is evidence that the malaria parasite can stimulate the production of haemolysin.^22^ Our observation is lower than those reported by studies conducted among blood group O donors in Lagos, Nigeria, in 2015 (18.6%)^10^ and Ilorin, Nigeria, in 2021 (31.7%).^11^ This may be due to differences in geographical location^26^ and the degree of exposure to gastrointestinal parasites^22^ and mosquitoes.^13,14,15^

Group O blood should not be transfused to blood group A, B, or AB recipients except when such blood is tested and determined to be free of haemolysins. The haemolysin test should be included in the protocol for screening and compatibility testing of blood donors in the National Blood Transfusion Programme of Cameroon. This can be achieved through the collaborative efforts of the government, the Ministry of Public Health of Cameroon, the National Blood Transfusion Programme of Cameroon, as well as staff of the RHB and RHBBB. Training for haemolysin testing should be conducted at the national level. Furthermore, more studies should be carried out in other localities in Cameroon to determine the haemolysin prevalence or presence of significant titres of haemolysin among blood group O donors.

### Limitations

Due to limited resources, we only used the visual titration method and could not carry out the spectrophotometric or gel methods for the quantification of red blood cell lysis; this could have influenced the detection of lysis. However, our findings were compared only with studies that used the visual method.

### Conclusion

Alpha and beta haemolysins are prevalent and exist in significant titres among blood group O donors in Bamenda, Cameroon. Thus, there is an urgent need for public health intervention. Considering the frequent practice of non-iso group-compatible ABO transfusion, there is a need to routinely test for the presence of haemolysins in blood donors to prevent the potential risk to recipients and to provide safer blood for maximum benefits to the recipient.
